# Ca^2+^ Signaling in Cardiac Fibroblasts and Fibrosis-Associated Heart Diseases

**DOI:** 10.3390/jcdd6040034

**Published:** 2019-09-23

**Authors:** Jianlin Feng, Maria K. Armillei, Albert S. Yu, Bruce T. Liang, Loren W. Runnels, Lixia Yue

**Affiliations:** 1 Calhoun Cardiology Center, Department of Cell Biology, University of Connecticut Health Center, Farmington, CT 06030, USA; feng@uchc.edu (J.F.); mkarmi20@colby.edu (M.K.A.); alyu@uchc.edu (A.S.Y.); bliang@uchc.edu (B.T.L.); 2 Department of Pharmacology, Rutgers, Robert Wood Johnson Medical School, Piscataway, NJ 08854, USA

**Keywords:** Ca^2+^ signaling pathways, TRP channels, cardiac fibroblasts, cardiac fibrosis, ion channels

## Abstract

Cardiac fibrosis is the excessive deposition of extracellular matrix proteins by cardiac fibroblasts and myofibroblasts, and is a hallmark feature of most heart diseases, including arrhythmia, hypertrophy, and heart failure. This maladaptive process occurs in response to a variety of stimuli, including myocardial injury, inflammation, and mechanical overload. There are multiple signaling pathways and various cell types that influence the fibrogenesis cascade. Fibroblasts and myofibroblasts are central effectors. Although it is clear that Ca^2+^ signaling plays a vital role in this pathological process, what contributes to Ca^2+^ signaling in fibroblasts and myofibroblasts is still not wholly understood, chiefly because of the large and diverse number of receptors, transporters, and ion channels that influence intracellular Ca^2+^ signaling. Intracellular Ca^2+^ signals are generated by Ca^2+^ release from intracellular Ca^2+^ stores and by Ca^2+^ entry through a multitude of Ca^2+^-permeable ion channels in the plasma membrane. Over the past decade, the transient receptor potential (TRP) channels have emerged as one of the most important families of ion channels mediating Ca^2+^ signaling in cardiac fibroblasts. TRP channels are a superfamily of non-voltage-gated, Ca^2+^-permeable non-selective cation channels. Their ability to respond to various stimulating cues makes TRP channels effective sensors of the many different pathophysiological events that stimulate cardiac fibrogenesis. This review focuses on the mechanisms of Ca^2+^ signaling in fibroblast differentiation and fibrosis-associated heart diseases and will highlight recent advances in the understanding of the roles that TRP and other Ca^2+^-permeable channels play in cardiac fibrosis.

## 1. Introduction

Cardiac fibrosis is involved in pathological remodeling of the heart, causing abnormalities in cardiac conduction, stiffness of the ventricular walls, reduced contractility, and impaired overall heart performance [1]. Thus, cardiac fibrosis is a detrimental factor in various types of heart diseases [2,3,4], including arrhythmia [5,6,7,8,9], hypertrophy [10], and heart failure [10,11,12,13]. There is substantial experimental evidence demonstrating that fibrosis slows down action potential propagation, initiates reentry, and promotes ectopic automaticity, thereby contributing to arrhythmogenesis [5,9,14]. Fibrosis also accelerates the progression of heart failure [15,16] resulting from nearly all etiologies of heart diseases, such as ischemic cardiomyopathy [17], dilated cardiomyopathy [18,19,20,21], hypertensive heart diseases [22,23], and inflammatory heart diseases [24]. Therefore, mitigating cardiac fibrosis by targeting the fibrogenesis cascade offers numerous promising therapeutic strategies for fibrosis-associated heart diseases [2,25].

**Figure 1:**
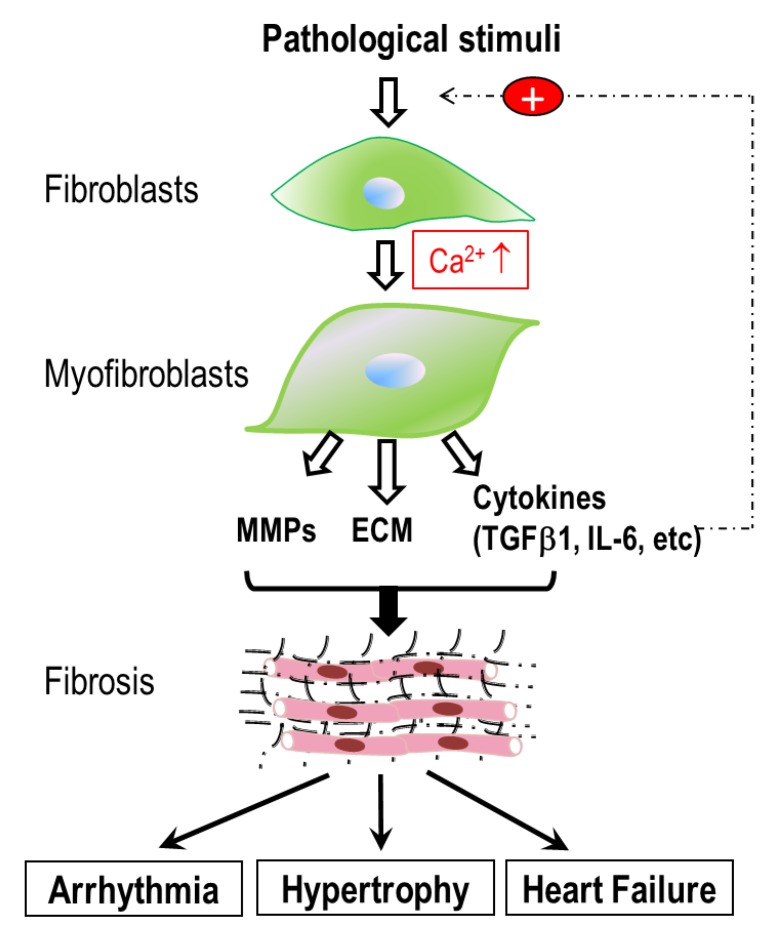
Schematic diagram illustrating cardiac fibrogenesis cascade and fibrosis-associated heart diseases. Pathological stresses stimulate fibroblasts to differentiate into myofibroblasts, during which an increase in intracellular Ca^2+^ plays a key role. Myofibroblasts synthesize and secrete extracellular matrix (ECM) proteins, matrix metalloproteinases (MMPs), and cytokines such as TGFβ1. Excessive deposition of ECM proteins results in cardiac fibrosis. This fibrogenesis cascade is perpetuated by TGFβ1 produced by myofibroblasts. Cardiac fibrosis is involved in a variety of pathological remodeling, which can lead to arrhythmia, hypertrophy and heart failure.

**Figure 2:**
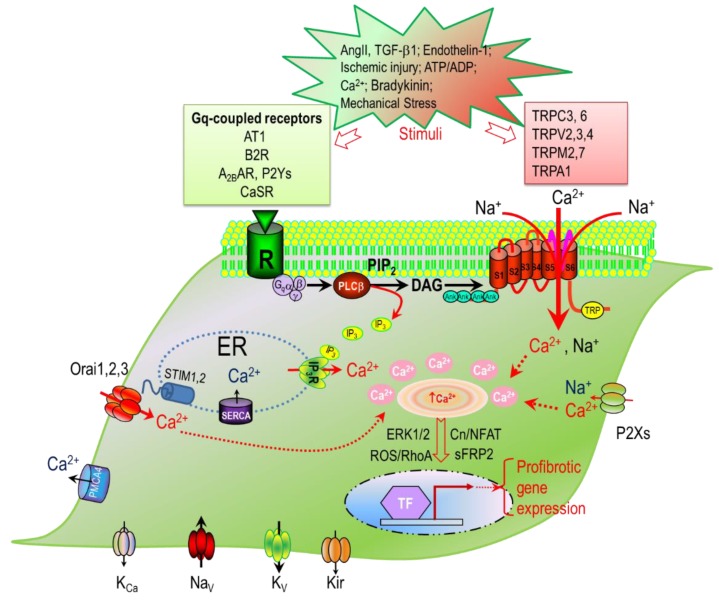
Ca^2+^ signaling mechanisms in cardiac fibroblasts and myofibroblasts. Intracellular Ca^2+^ levels are finely controlled by: (1) Ca^2+^ entry through Ca^2+^-permeable channels in the plasma membrane, including TRP channels, P2X receptors, and Orai/STIM channels; (2) Ca^2+^ release via IP_3_Rs in the ER; and (3) Ca^2+^ extrusion pumps, including SERCA in the ER and PMCA4 in the plasma membrane. Pathophysiological stimuli can activate Gq-coupled receptors to induce Ca^2+^ release, which is secondarily followed by Ca^2+^ entry. Receptor stimulation can also directly activate Ca^2+^-permeable (TRP) channels in the plasma membrane. Other ion channels, including voltage-gated K^+^ (K_v_) channels, Kir channels, Ca^2+^-activated potassium (K_Ca_) channels, and Na_V_ channels, may influence the resting membrane potential or depolarization to indirectly influence Ca^2+^ entry in fibroblasts and myofibroblasts. An increase in intracellular Ca^2+^ activates the calcineurin/NFAT (CN/NFAT), ERK1/2, ROS/RhoA, and sFRP2 pathways to promote profibrotic gene expression.

Fibrosis is the excessive accumulation of extracellular matrix (ECM) proteins synthesized by fibroblasts and myofibroblasts. The differentiation of fibroblasts into myofibroblasts is a pivotal step in the fibrogenesis cascade, as myofibroblasts are the predominant cell type that synthesizes and secretes ECM proteins [26]. Moreover, myofibroblasts produce growth factors, cytokines, and metalloproteinases, and are particularly responsive to proinflammatory cytokines, including tumor necrosis factor α (TNFα); interleukin-1 (IL-1), IL-6, and TGF-β; vasoactive peptide AngII, ET-1, ANP and BNP [27]; and hormones, such as noradrenaline. Thus, the differentiation of quiescent fibroblasts to active matrix-producing myofibroblasts is a key step in disease progression. A variety of pathological factors, such as oxidative stress, myocardial injury, unbalanced hormone levels, mechanical overload, and inflammatory stimuli can promote fibroblasts to differentiate into myofibroblasts. Increasing the differentiation of fibroblasts is, therefore, essential for initiating and perpetuating the fibrogenesis cascade [28,29,30,31,32,33,34] and the formation of fibrosis, which is involved in various pathological cardiac remodeling processes [9,35] (Figure 1). Numerous signaling pathways are involved in the activation of cardiac fibrogenesis [36], among which intracellular Ca^2+^ has been found to play a particularly critical role [37,38,39]. The Ca^2+^ signaling mechanisms in cardiac fibroblasts, however, are not fully understood. In recent years, there has been an increasing interest in elucidating the Ca^2+^ signaling mechanisms in cardiac fibroblasts and myofibroblasts. Similar to other cell types, Ca^2+^ signals in cardiac fibroblasts are generated by both Ca^2+^ release and Ca^2+^ entry. However, as non-excitable cells in the heart, cardiac fibroblasts are in a unique, complex environment, surrounded by multiple stimulating cues for fibrotic response. Recently, TRP channels have been found to be essential to fibroblast Ca^2+^ signaling. As Ca^2+^-permeable non-selective cation channels that respond to a variety of stimuli, TRP channels can not only directly initiate cellular Ca^2+^ signaling, but can also cause membrane depolarization to indirectly influence Ca^2+^ influx via other Ca^2+^-permeable channels. Moreover, although not gated by voltage, TRP channel activity can be influenced by membrane potentials. In addition, various fibrotic stimuli are known to activate Gq-coupled receptors in fibroblasts to induce Ca^2+^ influx through store-operated ion channels and TRP channels. This short review summarizes recent advances in the understanding of the different components contributing to intracellular Ca^2+^ signals, including Ca^2+^ release and Ca^2+^ entry, and their roles in cardiac fibrosis and fibrosis-associated heart diseases (Figure 2).

## 2. Ion Channels Controlling the Membrane Potential in Cardiac Fibroblasts

The membrane potential is a major force that controls Ca^2+^ entry in all different types of cells. Cardiac fibroblasts have a more depolarized resting membrane potential in comparison to cardiac myocytes. Measured by standard microelectrode techniques in multicellular tissues, the resting membrane potential of atrial fibroblasts was reported to be between –31 and –16 mV [40,41,42]. A similar resting membrane potential (about –37mV to –40 mV) was also obtained by patch-clamp recordings in isolated rat atrial fibroblasts [43,44]. The resting membrane potential was found to be more hyperpolarized in fibroblasts derived from canine heart failure models [44]. For example, it was increased from –43 mV in control fibroblasts to –56 mV in heart failure fibroblasts [44], pointing to the altered regulation of ion channels under diseased conditions [44]. Although fibroblasts are non-excitable cells, early studies have shown evidence that functional voltage-gated ion channels are expressed in cardiac fibroblasts [45]. Indeed, a growing number of studies have suggested the importance of both voltage- and non-voltage-gated ion channels in fibroblast function. 

### 2.1. K^+^ Channels

The expression of many different ion channel genes in cardiac fibroblasts has been detected at the RNA level by PCR and by patch-clamp current recordings in various studies (Table 1) [36,46]. Several voltage-gated ion channels and non-voltage-gated ion channels are present in cardiac fibroblasts (Table 1). For voltage-gated K^+^ channels, the transient-outward K^+^-current (I_to_), and delayed rectifier K^+^-currents have been demonstrated in rat [47,48], canine [49,50], and human [51] fibroblasts. These potassium channels are likely to be encoded by the α-subunits of voltage-gated K^+^ channel proteins, such as Kv1.2, Kv1.4, Kv1.5, and Kv2.1. In addition to the voltage-gated K^+^ channels, inward rectifier K^+^ (K_ir_ or I_K1_) currents have been recorded in rat [52], canine [53], and human [51] fibroblasts. I_K1_ activity has been reported to influence myofibroblast proliferation and contraction functions during in vitro experiments [52]. Upregulated I_K1_ has also been reported to play a role in atrial remodeling during atrial fibrillation in a canine model of congestive heart failure [53]. Another significant K^+^ conductance in fibroblasts is the ATP-activated potassium channel, K_ATP_ [51,52,54]. Activation of K_ATP_ in mouse cardiac fibroblasts increases cell proliferation, reduces IL-6 secretion [54], and inhibits fibroblast differentiation induced by ischemic injury in vitro [55]. Functional K_ATP_ expression in fibroblasts in vivo can be induced by myocardial infarction in the scar and border zone, which may influence the electrophysiological properties of myocytes [56]. Several types of Ca^2+^-activated K^+^ currents, including the big conductance (BK_Ca_) [51,57,58] and intermediate conductance (KCa3.1) [59], have been demonstrated in human and rat fibroblasts; these currents play a role in fibroblast proliferation [58], fibroblast-myocyte coupling [57], and the response to stretch [60].

### 2.2. Na^+^ Channels

Voltage-gated Na^+^ channels have been reported to be expressed in cardiac fibroblasts [51,58,61,62,63]. Both tetrodotoxin-sensitive and TTX-resistance Na^+^ currents (I_Na_) were recorded in cultured human ventricular fibroblasts [51]. In cultured human atrial fibroblasts, TTX-resistant I_Na_ was only identified in differentiated myofibroblasts, but not in freshly isolated fibroblasts [61]. The TTX-resistant I_Na_ exhibits similar properties to that of I_Na_ in cardiac myocytes, and is likely encoded by Na_V_1.5’s α-subunit and β1-subunit, as the expression levels of these two subunits were markedly higher in myofibroblasts than in fibroblasts [61]. Interestingly, although larger I_Na_ was found to present in a greater number of fibroblasts from atrial fibrillation patients than those from normal rhythm patients [63], I_Na_ did not appear to influence fibroblast proliferation, differentiation, or migration [61]. Moreover, as different α-subunits of Na^+^ channels are expressed in fibroblasts, including Nav1.2, Na_V_1.3, Na_V_1.5, Nav1.6, Nav1.7, and Na_V_1.9 [51,61,62], I_Na_ may be generated by a combination of different isoforms of the α subunits. Further investigation is required to understand the role of I_Na_ in fibroblasts’ and myofibroblasts’ functions and fibroblast-myocyte coupling. 

### 2.3. Cl^−^ and H^+^ Channels

Swelling-induced Cl^−^ currents [51,58] are present in human cardiac fibroblasts and are involved in fibroblast proliferation. Additionally, functional voltage-gated H^+^ currents are present in cardiac fibroblasts [66]. It was proposed that H^+^ currents in cardiac fibroblasts are involved in the regulation of intracellular pH and membrane potential under physiological conditions, as well as in the response to pathological conditions, such as ischemia, although their purpose has yet to be determined [66,74]. 

In summary, with growing interest in understanding the role of fibroblasts in cardiac physiology and pathology, the knowledge of cardiac fibroblasts’ electrophysiological properties will help elucidate how these properties influence the functions of cardiac fibroblasts, including the proliferation, differentiation, and secretion of ECM proteins, as well as the couplings of fibroblasts and myocytes, as reviewed recently [75]. The electrophysiological properties of fibroblasts can also directly influence Ca^2+^ signaling in fibroblasts, which plays a critical role in the cardiac fibrogenesis cascade. 

## 3. Ca^2+^ Signaling Mechanisms in Cardiac Fibroblasts

As a ubiquitous second messenger, Ca^2+^ is essential for nearly all cellular functions, including cell signaling, gene expression, cell proliferation, differentiation, migration, growth, and death [76,77]. Intracellular Ca^2+^ signals are generated by Ca^2+^ entry through Ca^2+^-permeable channels in the plasma membrane and Ca^2+^ release from intracellular Ca^2+^ stores. Intracellular Ca^2+^ levels are also finely controlled by plasma membrane ATPases (PMCAs), Na^+^/Ca^2+^ exchangers (NCX), and the sarcoplasmic reticulum Ca^2+^-ATPase (SERCA) [78]. Unlike cardiac myocytes, whose electrical properties and Ca^2+^ signaling mechanisms are well understood, the knowledge of Ca^2+^ signaling in cardiac fibroblasts is limited. In recent years, however, there have been extensive studies investigating the role of Ca^2+^ signaling in fibroblast physiology, and in fibrosis-associated heart diseases. 

### 3.1. Ca^2+^ Release Mechanisms in Cardiac Fibroblasts

There are several Ca^2+^ release mechanisms in different types of cells [78,79]. Ca^2+^ release from intracellular stores by ryanodine receptor (RyR) and IP_3_ receptor (IP_3_R) activation represent two major Ca^2+^ release mechanisms. There are also RyR-like Ca^2+^ release channels activated by cyclic ADP-ribose (cADPR) [78,79], as well as a distinct Ca^2+^-release pathway activated by nicotinic acid adenine dinucleotide phosphate (NAADP) [78,79]. NAADP is a potent intracellular Ca^2+^ release messenger originally identified in sea urchin lysates [80]. The target receptors of NAADP have been proposed to be RyR1 [81], and the two-pore channels (TPC) TPC1 and TPC2 [82,83,84,85]. However, whether TPC1 and TPC2 are the target receptors for NAADP is still controversial and requires further investigation [86].

Among the Ca^2+^ release pathways in the cardiac fibroblasts, no apparent role for RyRs has been demonstrated thus far. Although RyR blockers and activators have been reported to influence mechanically-induced potentials (MIP) recorded in fibroblasts in rat right atria [87], RyR expression, including that of RyR1, RyR2, and RyR3, cannot be detected by PCR in cultured human cardiac fibroblasts [46] or in neonatal rat cardiac fibroblasts [88]. Moreover, Ca^2+^ oscillations observed in cultured human cardiac fibroblasts are not influenced by ryanodine or caffeine [46]. Nonetheless, RyR2 expressed in myocytes has been shown to be involved in regulating TGF-β levels, thereby influencing fibrosis formation [88]. These results indicate that RyR may not have a direct role in fibroblasts under physiological and pathological conditions [88].

Similar to RyRs, which are known to be essential for the function of cardiac myocytes, the role of NAADP- and cADPR-induced Ca^2+^ signaling in cardiac myocytes has been demonstrated in various studies [89,90,91,92]. NAADP- and cADPR-mediated Ca^2+^ signaling contributes to the development of maladaptive cardiac hypertrophy induced by β-adrenergic stimulation [90]. However, although NAADP- and cADPR-mediated Ca^2+^ signaling has been shown to be crucial in various cell types, the role of these receptors in cardiac fibroblasts, if any, has not been determined.

Therefore, among different intracellular Ca^2+^ release pathways, IP_3_R activation-mediated Ca^2+^ signaling appears to be the major Ca^2+^ release pathway in cardiac fibroblasts. Chen and colleagues thoroughly investigated the expression of different components of Ca^2+^ release mechanisms in human cardiac fibroblasts, and found that all three types of IP_3_Rs are expressed in cultured human cardiac fibroblasts [46]. Moreover, Ca^2+^ oscillations can be completely blocked by the PLC blocker U73122 and the IP_3_R inhibitor 2-aminoethoxydiphenyl borate (2-APB). The IP_3_R agonist thimerosal can enhance Ca^2+^ oscillations [46]. Thus, it appears that IP_3_R activation-mediated Ca^2+^ release is the major Ca^2+^ release pathway in cardiac fibroblasts.

### 3.2. Signaling Pathways Mediating Ca^2+^ Release in Cardiac Fibroblasts

Many G-protein-coupled receptors (GPCRs) are expressed in cardiac fibroblasts [93]. AngII receptor AT1’s activation-induced intracellular Ca^2+^ increase has been proposed to be the signal transduction pathway responsible for AngII-mediated collagen synthesis [94]. Another Gq-coupled receptor activation by agonists has also been demonstrated to induce intracellular IP_3_ production in rat cardiac fibroblasts [95]. In addition to AngII, bradykinin (BK), ATP and UTP are also efficacious at inducing IP_3_ production, whereas endothelin-1 (ET1) and carbachol (100 μM) produce minimal or no change in IP_3_ production [95]. Accordingly, AngII (1 μM), BK (1 μM), UTP (30 μM), and ATP (30 μM) have been reported to induce substantial Ca^2+^, transiently, in rat cardiac fibroblasts, indicating that functional Gq-coupled receptors for AngII, BK, ATP, and UTP are present in cardiac fibroblasts [95]. 

The critical role of the AngII signaling pathway through fibroblast AT1 receptors in cardiac fibrosis has been well demonstrated [94,96,97,98]. Likewise, bradykinin receptors, Gi-coupled BR1, and Gq-coupled BR2 have been shown to be expressed in cardiac myofibroblasts [99,100]. In addition, purinergic signaling has also been shown to play an important role in cardiac fibrosis [101,102]. For the P1 receptors (adenosine receptors), RT-PCR has shown that mRNAs for all four P1 receptor subtypes, A_1_AR, A_2A_AR, A_2B_AR, and A_3_AR, are expressed in rat cardiac fibroblasts, with A_2B_ARs being the most abundant [102]. Activation of A_2B_AR, which is coupled to Gq and Gs, inhibits collagen synthesis, connective tissue growth factor (CTGF) expression, and fibroblast proliferation [103]. Moreover, overexpression of A_2B_ adenosine receptors results in a decrease in basal levels of collagen and protein synthesis, whereas silencing A_2B_ARs results in an increase in protein and collagen synthesis [102]. Activation of the P2Y receptors can also modulate fibroblast function. For example, agonists of P2Y2 receptors activate human cardiac fibroblast proliferation and migration [104]. Among the eight purinergic P2Y receptors (P2YRs) [101,105], five Gq-coupled receptors (P2Y1, P2Y2, P2Y4, P2Y6, and P2Y11) can be detected by RT-PCR, immunostaining, and a functional assay that measures inositol phosphate (IP) production in rat myofibroblasts [106]. 

Recently, it has been demonstrated that the calcium-sensing receptor (CaSR) is expressed in cardiac fibroblasts [107]. CaSR acts as a G-protein-coupled receptor that can induce an increase of intracellular Ca^2+^ via Gq-PLC activation. CaSR is expressed in various cell types, including cardiac myocytes, smooth muscle cells, neurons, and vascular endothelial cells. In cardiac fibroblasts, activation of CaSR increases intracellular Ca^2+^ concentration, promotes fibroblast proliferation and migration, and induces the synthesis of extracellular matrix proteins [107]. Moreover, the inhibition of CaSR reduced fibrosis in an isoproterenol-induced rat hypertrophy model [107]. Therefore, CaSR appears to be important for mediating Ca^2+^ release in cardiac fibroblasts (Figure 2).

### 3.3. Ca^2+^ Entry Mechanisms in Cardiac Fibroblasts 

Upon Gq-coupled receptor activation, Ca^2+^ release from intracellular stores is followed by Ca^2+^ entry through ion channels in the plasma membrane, which occurs through several different mechanisms. One pathway is mediated by store-operated Ca^2+^ entry (SOCE) through Ca^2+^ release-activated Ca^2+^ channels (CRAC). The second pathway occurs through non-selective Ca^2+^ entry after receptor activation, which is called receptor-operated Ca^2+^ entry (ROCE). ROCE is chiefly mediated by non-selective TRP channels [108], as discussed below. In addition to these two types of Ca^2+^ entry, purinergic P2X receptors (P2X1-7) can also directly mediate Ca^2+^ influx into cells. Although voltage-gated Ca^2+^ channel expression can be detected by PCR [46], functional currents have not been reported in cardiac fibroblasts.

#### 3.3.1. Voltage-gated Ca^2+^ Channels

Although it has been demonstrated that both voltage-gated Na^+^ channels and K^+^ channels are functionally expressed in cardiac fibroblasts, voltage-gated Ca^2+^ currents have not been identified. The expression of Ca_V_1.2 has been detected at the RNA expression level in human cardiac fibroblasts [46]. However, the functional voltage-gated Ca^2+^ channels cannot be obtained by patch-clamp [63]. Moreover, the Ca^2+^ channel blocker nifedipine and activator Bay K8644 failed to affect Ca^2+^ oscillations in cardiac fibroblasts, suggesting that L-type Ca^2+^ channels are not involved in Ca^2+^ signaling in this cell type [46]. Interestingly, a potential role for the voltage-gated Ca^2+^ channels has been suggested in lung fibrosis, since nifedipine inhibits Ca^2+^ oscillations and attenuates bleomycin-induced lung fibrosis [109]. These findings suggest that fibroblasts in different organs and tissues may have varied molecular components and exhibit distinct properties. 

#### 3.3.2. Ca^2+^ Entry Mediated by P2X Receptors

Another type of Ca^2+^-permeable channel identified in cardiac fibroblasts is the family of ATP-activated Ca^2+^-permeable non-selective cation channels, P2Xs, or P2X receptors (P2XR: P2X1-7). P2X receptors are widely distributed in excitable and nonexcitable cells [110]. The non-selective cation permeation of P2XRs not only brings Ca^2+^ into the cells, but also causes depolarization. Among the seven members of the P2XR family, P2X7 is present in human primary skin fibroblasts [111]. P2X7 has been shown to be involved in tissue fibrosis [112,113]. Since P2X7 is one of the major mediators of inflammasome activation, the mechanisms by which P2X7 activation causes fibrosis are unclear. In cardiac fibroblasts, P2X4 and P2X7 mRNAs have been detected in cultured human ventricular fibroblasts [46]. Furthermore, cardiac myocyte-specific overexpression of the P2X4 receptor in mice decreases fibrosis in a hypertensive heart failure mouse model [114,115], yet it is unknown whether this is due to direct cardiac myocyte protection or the interaction between cardiac myocytes and fibroblasts [116]. The function of P2X4 and P2X7 in mediating Ca^2+^ entry in cardiac fibroblasts and their role in cardiac fibrosis needs further investigation. 

#### 3.3.3. Ca^2+^ Entry through CRAC (Orai/STIM) Channels in Cardiac Fibroblasts

The Ca^2+^ release-activated Ca^2+^ channel (CRAC) is essential for cellular functions in a variety of cells [117,118,119]. The molecular components of CRAC channels consist of the pore-forming subunit Orai (Orai1, Orai2, and Orai3), and the Ca^2+^ release-sensing subunit STIM (STIM1 and STIM2). The role of Orai/STIM in cardiac myocytes and cardiac function has been extensively studied [120,121,122,123]. However, controversial results have been reported regarding the contribution of Orai/STIM to cardiac physiology and pathology [120,124]. In fibroblasts, although the Orai and STIM subunits have been detected by qPCR or RT-PCR in mouse and human cardiac fibroblasts [37,46], CRAC currents have not been recorded by patch-clamp [37]. Recently, the functional Orai1/STIM1 channels have been demonstrated by Ca^2+^ imaging measurements in human ventricular fibroblasts [67,68,125]. Ca^2+^ influx, and the expression of Orai1 but not STIM1, have been shown to be much larger in ventricular fibroblasts derived from failing hearts compared to those of non-failing hearts [68]. In aged ventricular fibroblasts, while store-operated Ca^2+^ release and entry were shown to increase, the expressions of Orai1 and STIM1 remained unchanged [67]. These results suggest that Orai/STIM channels play a role in fibroblast Ca^2+^ signaling, and may therefore contribute to cardiac remodeling under pathological conditions. Nonetheless, further investigations using the fibroblast-specific deletion of Orai/STIM proteins may help to clarify the physiological and pathological roles of Orai/STIM in the heart.

#### 3.3.4. Ca^2+^ Entry Mediated by TRP Channels in Cardiac Fibroblasts and Fibrotic Heart Disease

##### Overview of TRP Channels

Transient receptor potential (TRP) channels belong to a superfamily of non-voltage-gated Ca^2+^-permeable ion channels that can be divided into six subfamilies, including the TRPC (canonical), TRPV (vanilloid), TRPM (melastatin), TRPA (ankyrin), TRPP (polycystin), and TRPML (mucolipin) subfamilies [126,127]. There are different members in each subfamily; for example, the TRPC, TRPV, and TRPM subfamilies contain seven (TRPC1-7), six (TRPV1-6), and eight (TRPM1-8) members, respectively [126,127,128]. The TRPA subfamily has one channel protein [129,130,131]; the TRPP and TRPML subfamilies contain three channel proteins each [127,132]. The TRPP (polycystin) and TRPML (mucolipin) subfamilies are intracellular ion-channels [133], whereas the other TRP subfamilies appear to function at the plasma membrane. Most TRP channels are Ca^2+^-permeable non-selective cation channels, with the exceptions of TRPM4 and TRPM5, which are monovalent cation-selective channels [126,127], and TRPV5 and TRPV6, which are highly Ca^2+^-selective (*P*_Ca_/*P*_Na_>100) [126,127,134,135]. TRPM6 and TRPM7 are unique channel proteins because they have a kinase domain at their C-terminals. Both TRPM6 and TRPM7 are permeable to Ca^2+^, Mg^2+^, Zn^2+^, and other trace metals [72,136,137,138]. 

##### TRP Channels are Multifunctional Cellular Sensors

TRP channels are not gated by voltage, but rather, these channels are responsive to a wide range of stimuli in a polymodal activation manner, including thermal, mechanical, oxidative, chemical, and nociceptive stresses, and local autocrine or paracrine environmental cues [127,139]. TRPV1–TRPV4 and TRPM3 are activated by high temperatures, whereas TRPM8, TRPA1, and TRPC5 are turned on at low temperatures [140,141,142]. Many of the TRPC channels, including TRPC2 [143], TRPC3 [144], TRPC6 [144], and TRPC7 [145], are activated by Gq-linked receptor activation; meanwhile, some of the TRPCs can be directly activated by diacylglycerol (DAG) [146]. Different TRPCs can form heterotetrameric channels, such as the TRPC1/TRPC4/TRPC5 and TRPC3/TRPC6/TRPC7 functional channels [147]. The Mg^2+^-permeable TRPM6 and TRPM7 are activated by lowering the intracellular free Mg^2+^ concentration [136,137,148,149,150], and these channels are likely to be constitutively active to a small degree under physiological conditions. Changes in intracellular Ca^2+^ ([Ca^2+^]_i_) influence the activation of many TRP channels. A rise of intracellular Ca^2+^ ([Ca^2+^]_i_) activates several TRP channels, including TRPM4 [151], TRPM5 [152,153], TRPM2 [154], and TRPA1 [155]; in contrast, a decrease of intracellular Ca^2+^ ([Ca^2+^]_i_) can activate TRPV5 and TRPV6. TRPM2 can also be activated by multiple stimuli, including [Ca^2+^]_i_, ADP-ribose (ADPR), NAD^+^, and oxidative stress [156,157,158]. The polymodal activation feature of TRP channels makes them extremely powerful effectors for integrating and responding to a range of physiological and pathological stimuli, which in turn can trigger various pathogenesis cascades. 

##### The Functional Expressions of the TRP Channels in Cardiac Fibroblasts

Many TRP channels have been detected at the RNA level by qPCR or RT-PCR in the cardiac fibroblasts of various species [37,46,159]. TRPC1, TRPC2, TRPC3, TRPC5, TRPC6, and TRPC7 are present in isolated rat fibroblasts [69,160]; TRPC1, TRPC4, and TRPC6 are expressed in cultured human cardiac fibroblasts [46]; and TRPC1, TRPC6, TRPV2, TRPV4, and TRPM7 can be detected in freshly isolated human atrial fibroblasts by RT-PCR [37]. In mouse cardiac fibroblasts, TRPC1, TRPC3, TRPC4, TRPC6, TRPV2, TRPV4, TRPM4, TRPM6, and TRPM7 are abundantly expressed, as assessed by RT-PCR. Moreover, all the TRP channel transcripts have been detected by qPCR in isolated mouse cardiac fibroblasts [37]. Although the expression of TRP channels can be readily detected by RT-PCR, only a few TRP channels can be functionally detected by patch-clamp current recording, as summarized in Table 1.

The first TRP channel current recorded in cardiac fibroblasts was the TRPM7 current recorded in rat cardiac fibroblasts [72]. TRPM7 currents can also be readily recorded in other fibroblasts, such as in human and mouse cardiac fibroblasts [37]. The TRPM7-like currents recorded in mouse and human fibroblasts exhibit similar biophysical and pharmacological properties to those of the heterologously-expressed TRPM7 currents [137,149,161,162], including sensitivity to external acidic pHs, and a distinct response to 2-APB in comparison to TRPM6. TRPC3, TRPC6, or TRPC3/6 heteromer-like currents have been reported in rat ventricular fibroblasts [69], and TRPC3-like currents have also been recorded in rat atrial fibroblasts [70]. Similarly, a TRPM2-like current has been recorded in rat cardiac fibroblasts after 24 hours of H_2_O_2_ treatment, suggesting hypoxia up-regulates TRPM2 in rat cardiac fibroblasts [59]. The TRPM2-like current can be inhibited by Clotrimazole, and reduced by TRPM2 siRNA treatment. Using 4α-12,13-didecanoate (4α-PDD) as an activator, TRPV4-like currents have been recorded in rat cardiac fibroblasts. The 4α-PDD-induced TRPV4 current is blocked by ruthenium red, and is reduced by TRPV4 siRNA [71]. Thus, many different TRP channels are expressed in cardiac fibroblasts, and recent work is adding to a growing body of evidence that TRP channels in cardiac cells, including myocytes and fibroblasts, play an essential role in heart diseases [159,163]. In this review, we focus on summarizing the recent advances regarding the role of TRP channels in mediating Ca^2+^ signaling in cardiac fibroblasts, and their potential roles in cardiac fibrosis and fibrosis-associated heart diseases [37,39,70,164,165] (Table 2).

### 3.4. Ca^2+^ Signaling Mediated by TRP Channels in Cardiac Fibroblasts and Fibrosis-Associated Heart Diseases

#### 3.4.1. TRPC Channels

The role of TRPC channels in hypertrophy and heart failure has been extensively studied by the systematic or myocardial knockdown, overexpression, and knockout of TRPC channels, as previously reviewed [159,163,165,201]. Most of the TRPC channels, TRPC1, TRPC3, TRPC6, TRPC7, and the heterotetrameric channel complexes TRPC3/6/7, TRPC1/4/5, and TRPC1/TRPC4 [202], have been demonstrated to be important mediators of pathological hypertrophy, and may serve as therapeutic targets [201,203]. Indeed, the TRPC3 blocker Pyrazole-3 (Pyr3) [166], the combined TRPC3 and TRPC6 channel blockers GSK2332255B and GSK2833503A [204], and the TRPC6 channel blocker BI-749327 [38], are effective at attenuating pathological remodeling and improving heart function. Although these studies provide strong evidence that TRPC channels in cardiac myocytes play an essential role in mediating hypertrophic remodeling, it remains unclear whether TRPC channels in cardiac fibroblasts contribute to pathological remodeling.

Despite knowledge of the role of TRPC channels in fibroblasts in hypertrophic remodeling being limited, it has been shown that the pathological stimuli AngII and ET1 can indeed induce Ca^2+^ entry through TRPC3 [70] and TRPC6 [160], respectively. Moreover, nucleotides released during ischemic injury activate P2Y2 and P2Y4 receptors to cause Ca^2+^ release and subsequent Ca^2+^ entry via TRPC channel activation, which leads to fibroblast differentiation [205]. Nonetheless, the role of TRPC3 and TRPC6 in fibrogenesis has been investigated in great detail recently [70,166].

TRPC3 and Fibrosis-Associated Arrhythmia and Heart Failure

TRPC3 channels have been shown to play a role in cardiac fibrosis and fibrosis-associated heart diseases, such as atrial fibrillation (AF) [70] and heart failure induced by pressure overload [166,167]. It has been recently demonstrated that TRPC3, independent of TRPC6, mediates pressure-overload-induced maladaptive cardiac fibrosis [168,169]. Pharmacological inhibition of TRPC3 suppresses the fibrotic response in human cardiac myocytes and fibroblasts [169]. Administration of the combined TRPC3 and TRPC6 blocker GSK503A to pressure-overload mice or rats also results in antifibrotic effects [204]. In pressure-overload mouse hearts, the inhibition of TRPC3 reduces RhoA-mediated maladaptive fibrosis [169]. It was proposed that TRPC3-mediated intracellular Ca^2+^ signaling activates PKC, which then phosphorylates p47^phox^ and activates NADPH oxidase 2 (Nox2) to generate ROS, thereby initiating the RhoA signaling pathway and ECM production [167,168,169]. 

TRPC3-mediated Ca^2+^ signaling in fibroblasts is also involved in arrhythmogenesis [70]. TRPC3 is upregulated in the atria of atrial fibrillation (AF) patients, and in atrial fibrillation goat, and dog models [70]. The mechanism by which atrial fibrillation increases TRPC3 expression is mediated by NFAT-induced downregulation of microRNA-26 [70]. Enhanced TRPC3 expression increases fibroblast proliferation and differentiation, likely by controlling Ca^2+^ influx, which activates extracellular signal-regulated kinase (ERK1/2) signaling [70]. TRPC3-mediated fibroblast proliferation and differentiation through the ERK signaling pathway has also been reported in other studies [170]. In vivo administration of the TRPC3 blocker Pyr3 reduces ECM protein expression and suppresses development of the AF substrate in the electrically-maintained dog model of atrial fibrillation [70]. Thus, reducing TRPC3-mediated Ca^2+^ signaling appears to reduce the susceptibility to AF, perhaps by reducing fibroblast cell proliferation. Overall, the data suggest that TRPC3 is likely a potential therapeutic target for fibrosis-associated atrial fibrillation [70].

TRPC6-Mediated Fibroblast Differentiation and Heart Failure

TRPC6 has been reported to regulate fibroblast differentiation induced by ET1 in rat ventricular fibroblasts [160]. TRPC6-mediated Ca^2+^ influx by ET1 in neonatal rat ventricular fibroblasts activates NFAT, which acts as a negative regulator of ET1-induced fibroblast differentiation [160]. In contrast, other studies report that TRPC6 instead promotes fibroblast proliferation and differentiation [171]. It has also been demonstrated that TRPC6-mediated Ca^2+^ signaling is required for human cardiac fibroblast proliferation induced by AngII and OAG (1-oleoyl-2-acetyl-sn-glycerol); the latter is a diacylglycerol (DAG) analogue [171]. Consistent with a positive role for TRPC6 in fibroblast differentiation, Davis and colleagues have demonstrated that TRPC6 is necessary and sufficient for fibroblast differentiation induced by AngII [164]. It has been shown that TRPC6 is upregulated by TGF-β1 and AngII via the p38 MAPK (mitogen-activated protein kinase) serum response factor [164]. Activation of TRPC6 stimulates the calcineurin/NFAT pathway to induce fibroblast differentiation [164]. Fibroblasts lacking TRPC6 (TRPC6^-/-^) were not able to differentiate into myofibroblasts in response to TGF-β1 stimulation [164]. Also, mice without TRPC6 displayed impaired dermal and heart-wound healing function [164]. Consistent with the role of TRPC6 in promoting fibroblast differentiation, silencing TRPC6 with a siRNA approach attenuated the TGF-β1-mediated upregulation of α-SMA in the human right ventricle [172]. Moreover, administration of the TRPC6 antagonist (BI-749327) to mice subjected to pressure-overload inhibited the profibrotic gene expression, reduced cardiac fibrosis, and improved heart function [38], suggesting that inhibition of TRPC6 in fibroblasts at least partially mediates the protective effects in the attenuation of pressure-overload-induced cardiac remodeling.

#### 3.4.2. TRPV Channels in Fibroblasts and Cardiac Fibrosis

Several TRPV channels have been implicated in cardiac fibrosis, including TRPV1, TRPV2, TRPV3, and TRPV4. We summarize their putative roles below.

TRPV1 and Cardiac Fibrosis

TRPV1 is predominately expressed in peripheral sensory neurons and is widespread in the cardiovascular system [206]. The effects of TRPV1 in cardiac fibrosis have been controversial. Some studies demonstrate that deletion of TRPV1 results in stimulation of the TGF-β1 and SMAD2 signaling pathway, and therefore, significantly increases fibrosis in a myocardial injury model [207]. In contrast, another study reported that the activation of TRPV1 by capsaicin blunts pressure-overload-induced hypertrophy and fibrosis [173]. It has also been shown that capsaicin reduces fibroblast proliferation induced by AngII in vitro [173]. Moreover, overexpression of TRPV1 in transgenic mice attenuates isoproterenol-induced myocardial fibrosis [174], and the activation of TRPV1 has also been shown to be protective in a myocardial injury model [175,176,177]. On the contrary, other studies demonstrate that administration of a TRPV1 antagonist BCTC (4-(3-Chloro-2-pyridinyl)-N-[4-(1,1-dimethylethyl)phenyl]-1-piperazinecarboxamide) prevents the loss of heart function, and protects the heart from fibrosis in a pressure-overload mouse model [208]. Mice lacking TRPV1 are also protected from pressure-overload-induced cardiac hypertrophy [209]. The cause of these discrepancies in different studies regarding the role of TRPV1 in fibrogenesis is unclear, but it could be attributed to the expression of TRPV1 in different cell types and the channel’s involvement in different signaling pathways.

TRPV2 and Cardiac Fibrosis

TRPV2 has been shown to play a role in Ca^2+^-induced myocyte degeneration in dilated cardiomyopathy [178]. A knockout of TRPV2 protects hearts against pressure-overload-induced hypertrophy, but produces no protection against AngII or β-adrenergic activation-induced hypertrophy, indicating that TRPV2 regulates cardiac hypertrophy through stretch activation [179]. A knockout of TRPV2 in mice also reduces age-related fibrosis and hypertrophy [210]. Application of the TRPV2 blocker Tranilast blunts the hypertrophic and fibrotic response to pressure-overload-induced hypertrophy within four weeks [180]. Although TRPV2 has been shown to be located intracellularly and translocates to the sarcolemma under stress, the effect of Tranilast does not seem to be mediated through its effects on myocytes [180]. TRPV2 is also expressed in other cell types, such as macrophages, and in fact, TRPV2 knockout improves heart performance after myocardial infarction due to attenuated activity of peri-infarct macrophages [181]. TRPV2 is also highly expressed in cardiac fibroblasts and has been shown to regulate dermal fibroblast differentiation [211]. Thus, it is plausible that TRPV2-mediated Ca^2+^ signaling in cardiac fibroblasts regulates fibroblast differentiation and cardiac fibrosis. Further studies are required to dissect the different roles of TRPV2 during heart injury.

TRPV3 and Cardiac Fibrosis

TRPV3 is expressed in the peripheral neurons and various other cell types. It has recently been found that TRPV3 is also expressed in cardiac myocytes [182] and fibroblasts [183], as detected by PCR and western blotting (WB). In rat cardiac fibroblasts, the activation of TRPV3 by Carvacrol increases cell proliferation and upregulates the expression of collagen and TGF-β1. In a pressure-overload hypertrophy rat model, Carvacrol activation of TRPV3 exacerbates heart function and increases fibrosis [182,183]. These results suggest that TRPV3 could be involved in TGF-β1-induced fibroblast proliferation during cardiac fibrosis, but additional experiments with knockout mice are required to conclusively define the channel’s role.

TRPV4 and Cardiac Fibrosis

TRPV4 has been shown to regulate fibroblasts’ differentiation into myofibroblasts by integrating TGF-β1 signals and mechanical stimulation [39,184]. In cultured rat fibroblasts, TRPV4 agonists elicit a substantial Ca^2+^ influx. The application of a TRPV4 antagonist and an shRNA knockdown of TRPV4 inhibits TGF-β1-induced myofibroblast differentiation, whereas TGF-β1-treated fibroblasts exhibit enhanced TRPV4 expression and increased Ca^2+^ influx [39]. In a lung fibrosis mouse model, it has been shown that deletion of TRPV4 protects mice from lung fibrosis [212]. Similarly, in a myocardial infarction model, TRPV4 deletion in mice also reduces fibrosis and protects the heart from pathological remodeling [185]. These studies highlight an important role for TRPV4 in cardiac fibrosis, making it a potentially attractive therapeutic target.

#### 3.4.3. TRPM Channels and Cardiac Fibrosis

Several TRPM channels have been shown to play important physiological or pathological roles in the heart. TRPM2 has been found to be involved in ischemic cardiomyopathy, with protective roles reported by some studies [186,187], and exacerbated roles reported by others [188,189]. In cardiac fibroblasts, TRPM2 current can be induced in rat fibroblasts after treatment with H_2_O_2_ [59]. However, whether TRPM2 channel activity influences fibroblast function remains unknown. The role of TRPM4 in cardiac fibroblasts has not been evaluated, although TRPM4 channel function has been shown to be essential for cardiac conduction, and the dysfunction of TRPM4 is associated with conduction defects and arrhythmia [213,214,215,216]. 

Physiological Function of TRPM7 in the Heart

TRPM7 is essential for embryogenesis [217], organogenesis [218], and cardiogenesis [190,191]. The global deletion of TRPM7 in mice results in embryonic lethality before embryonic day 7 (E7). Myocyte-specific deletion of TRPM7 before E9 results in impaired compact myocardium development with consequential congestive heart failure and embryonic death by E11.5 [190]. However, myocyte deletion of TRPM7 at about E13 produces viable mice with normal adult ventricular sizes and heart function [190], indicating that TRPM7 is dispensable for the adult mice [190]. However, if TRPM7 is deleted in myocytes between day E9.5 and E12.5, 50% of mice have normal heart function, and another 50% of mice exhibit penetrant adult cardiomyopathy characterized by ventricular dysfunction, hypertrophy, fibrosis, disputed atrioventricular conduction, dispersed ventricular repolarization, and ventricular arrhythmia [190]. These results indicate that TRPM7 deletion in myocytes during the developmental stage (E9.5 to E12.5) can cause impaired adult heart function, whereas deletion of TRPM7 after embryonic day 13 does not produce any effects on normal heart function [190]. Interestingly, a recent study by Rios and colleagues reported that TRPM7-deficient mice with kinase domain deletion (TRPM7^+/^^Δkinase^) exhibit cardiac hypertrophy, fibrosis, and inflammation [219]. It was suggested that TRPM7 plays both anti-fibrotic and anti-inflammatory roles [219]. However, since TRPM7 channel activity was impaired in the TRPM7^+/^^Δkinase^ mice [220], it is plausible that the disrupted TRPM7 channel function during the developmental stage (E9.5 to E12.5) may have contributed to the impaired heart function, resulting in the observed cardiac hypertrophy, inflammation, and fibrosis in the TRPM7^+/^^Δkinase^ mice [219].

Expression of TRPM7 in Cardiac Fibroblasts

TRPM7 is also highly expressed in cardiac fibroblasts. Endogenous TRPM7-like currents were initially recorded in rat cardiac fibroblasts [72], and were readily recorded in mouse and human fibroblasts [37,221,222]. TRPM7-like current is significantly upregulated in fibroblasts from atrial fibrillation patients [37]. Although TRPM6 expression was found to be upregulated in the right atria of atrial fibrillation patients in comparison with sinus rhythm patients [223], the endogenous currents recorded in human atrial fibroblasts are encoded by TRPM7, as TRPM7 siRNA eliminates the majority of TRPM7-like currents. TRPM6 and TRPM7 can form heteromeric channels [137,149,161]. The homomeric TRPM6 and TRPM7 channels and heteromeric TRPM6/7 channels share similar biophysical properties, such as current-voltage (I-V) relation, the potentiation for inward current by externally low pH, and regulation by PIP_2_, but they have distinct single-channel conductances and pharmacological sensitivities to low and high concentrations of 2-APB [137,149,161,224]. The endogenous TRPM7-like currents recorded in human atrial fibroblasts display a similar single-channel conductance and 2-APB sensitivity to that of TRPM7 currents recorded in the over-expression system, indicating that the functional current in human atrial fibroblasts is encoded by TRPM7. It will be of interest to investigate whether TRPM6 contributes to functional expression of TRPM7-like currents in cardiac fibroblasts.

TRPM7 and Fibroblast Differentiation

TRPM7-mediated Ca^2+^ entry is significantly enhanced in fibroblasts from AF patients. Knockdown of TRPM7 inhibits TGF-β1-induced fibroblast proliferation, differentiation, and collagen production [37], whereas upregulation of TRPM7 by TGF-β1 in cultured human atrial fibroblasts or mouse cardiac fibroblasts enhances fibroblast proliferation, differentiation, and ECM production [37]. It appears that TRPM7-mediated Ca^2+^ signaling is essential for TGF-β1-induced fibroblast proliferation, differentiation, and the fibrogenesis cascade [37]. A role of TRPM7 in mediating the fibrogenesis cascade has also been observed in rat cardiac fibroblasts [193,194,195,196]. Moreover, TRPM7 is also suggested to be involved in fibrogenesis in rat sinus nodes [192], and in fibrosis induced by isoproterenol and oxidative stress [195]. Thus, it seems that TRPM7 may serve as a potential target for fibrosis-associated heart diseases.

#### 3.4.4. TRPA1 in Cardiac Fibroblasts

TRPA1 is expressed in cardiac myocytes [197,198] and fibroblasts [199]. Activation of TRPA1 elicits Ca^2+^ influx in primary cultured human ventricular cardiac fibroblasts [73]. TRPA1 selective inhibitors HC-030031 (HC) and TCS-5861528 (TCS) ameliorate pressure-overload-induced cardiac hypertrophy by negatively regulating Ca^2+^/calmodulin-dependent protein kinase II (CaMKII) and calcineurin signaling pathways [200]. The knockout TRPA1 increases survival rate, reduces fibrosis, and enhances heart performance after myocardial infarction [199]. Moreover, in primary cultured cardiac fibroblasts, an overexpression of TRPA1 potentiates, whereas a knockdown TRPA1 attenuates, TGF-β1-induced fibroblast differentiation. Finally, it appears that TRPA1 regulates fibroblast differentiation through the calcineurin-dependent NFAT3 activation pathway [200]. 

### 3.5. Ca^2+^ Efflux and Fibroblast Function

In contrast to the sarcoplasmic reticulum (SR) Ca^2+^-ATPase (SERCA) and the plasma membrane Na^+^/Ca^2+^ exchanger (NCX), the two major components that extrude cytosolic Ca^2+^ to the SR/ER and outside of the cell, the plasma membrane Ca^2+^-ATPase (PMCA1,2,3,4) was considered to be relatively insignificant in maintaining Ca^2+^ homeostasis in the heart [225,226]. In recent years, however, it has been demonstrated that PMCA4 plays a significant role in the regulation of signal transduction in the heart. 

Among the four PMCAs, the expression of PMCAs 1, 3, and 4 has been detected at the mRNA level in human cardiac fibroblasts [46]. Recently, it has been shown that the deletion of PMCA4 in mouse fibroblasts, but not in myocytes, protects the heart against hypertensive hypertrophy [227]. The basal Ca^2+^ level in PMCA4-KO fibroblasts is 25% higher than that in control fibroblasts. PMCA4 ablation also increases secreted, frizzled-related protein 2 (sFRP2) expression, which inhibits the hypertrophic response in myocytes [227]. Moreover, the PMCA4 inhibitor aurintricarboxylic acid (ATA) has been reported to inhibit and reverse cardiac hypertrophy induced by pressure-overload in mice [227]. These results indicate that regulating Ca^2+^ homeostasis in fibroblasts can also mitigate fibrosis-associated heart diseases.

NCX and SERCA are essential to maintaining Ca^2+^ homeostasis in many cell types, such as cardiac myocytes. In cardiac fibroblasts, the mRNA expression of NCX and SERCA has also been detected in human ventricular fibroblasts [46]. It was suggested that NCX plays a role in Ca^2+^ homeostasis in fibroblasts [46], based on the effects of the NCX inhibitor, Ni^2+^, and extracellular Na^+^ replacement, on Ca^2+^ oscillations in human ventricular fibroblasts. However, the detailed characterization of NCX and SERCA in cardiac fibroblasts, and their roles in fibroblast function and in cardiac fibrosis, still remain largely unknown.

## 4. Conclusions and Future Perspectives

Fibrosis is a hallmark feature of most heart diseases. There are a multitude of signaling pathways, bioactive molecules, and various cell types that are involved in the cardiac fibrogenesis cascade. As the major cell type in the fibrogenesis cascade, fibroblasts are the receivers of various pathological stimuli and the producers of extracellular matrix proteins. However, the Ca^2+^ signaling mechanisms controlling cardiac fibroblast functions are not fully understood. Nevertheless, the advances in recent years have helped to shape our understanding of the complex nature of Ca^2+^ signaling mechanisms in fibroblasts, while TRP channels with their unique features have emerged as the most important ion channels that mediate Ca^2+^ signals in cardiac fibroblasts. Evidence suggests that many TRP channels have the potential to be therapeutic targets for drug intervention. However, more work is necessary to understand TRP channels’ functions in the heart. As TRP channels are widely expressed in different cell types, future investigations using cell type-specific deletions of TRP channel genes will provide more precise information regarding their specific roles in cardiac fibroblasts. Moreover, as in vitro fibroblast experiments are an important part of cardiac fibroblast research, finding the experimental conditions that closely mimic the in vivo properties of fibroblasts and myofibroblasts will be critical. Finally, since fibrogenesis involves multiple cell types in the heart, gaining a better understanding of the fibroblast differentiation process, and how Ca^2+^ signaling influences the interactions of fibroblasts and myofibroblasts with myocytes and other cell types, is an important challenge whose outcomes will provide new insights into the therapeutic potential of TRP channels in fibrosis-associated heart diseases. 

## Figures and Tables

**Table 1:** Membrane currents in cardiac fibroblasts.

Types of Ion Channels	Names of the Ion Channels	Cell Types Expressing the Ion Channels
Voltage-gated Na^+^ currents	I_Na.TTX_, I_Na.TTXR_, [51]; I_Na.TTXR_ [61] Na_V_1.2, Na_V_1.9 [62]	Human cardiac fibroblasts [51,63]
Voltage-gated K^+^ channels	Transient outward K^+^ current, I_to_ [51] [47,64]	Human cardiac fibroblasts [51], neonatal rat cardiac fibroblasts [47,64]
	Delayed rectifier K^+^ currents: I_K_ [48,52], I_Kf_ and I_KS_ [47], IK_DR_ [51]	Neonatal [47,64] and adult rat cardiac fibroblasts [48,52]; human cardiac fibroblasts [51]
Inward-rectifier K^+^ channels	Inward-rectifying K^+^ currents: K_ir_ [51,52], I_K1_ [44,53]	Human cardiac fibroblasts [51], adult rat cardiac fibroblasts [52], dog atrial fibroblasts [44,53]
ATP-activated K^+^ channels	K_ATP_ [54,56]	Rat ventricular fibroblasts [54,56]
Ca^2+^-activated K^+^ channels	Big conductance K^+^ currents activated by Ca^2+^: BK_Ca_ [51,57]	Human cardiac fibroblasts [51,57]
	Intermediate conductance K^+^ currents activated by Ca^2+^: KCa3.1 [59], I_KCa_ [65]	Rat adult cardiac fibroblasts [59,65]
Cl^−^ channels	Swelling-induced Cl^−^ currents, I_Cl-swell_ [51]	Human cardiac fibroblasts [51]
Voltage-gated H^+^ channels	Voltage-gated H^+^-currents [66]	Human cardiac fibroblasts [66]
Store-operated Ca^2+^ channels (Orai/STIM)	Ca^2+^ release-activated Ca^2+^ currents: I_CRAC_ [67,68]	Human ventricular fibroblasts [67,68]
TRP channels	TRPC-like currents (TRPC3 or TRPC6) [69]	Adult rat ventricular fibroblasts [69]
	TRPC3 [70]	Rat atrial fibroblasts [70]
	TRPV4 [71]	Rat cardiac fibroblasts [71]
	TRPM2 [59]	Rat adult fibroblasts [59]
	TRPM7 [37,72]	Mouse, rat, and human cardiac fibroblasts [37,72]
	TRPA1 [73]	Human cardiac fibroblasts [73]

**Table 2:** Potential role of Ca^2+^-permeable TRP channels in cardiac fibroblasts and fibrosis-associated heart diseases.

Channels	Expression in Cardiac Cells	Cellular Function in Fibroblasts	Methods Used for In Vivo Pathological Functions	Potential Roles in Heart Diseases	References
TRPC3	Myocytes, fibroblasts	Proliferation, differentiation	Specific blockers	Arrhythmia, hypertrophy, heart failure	[70,166,167,168,169,170]
TRPC6	Myocytes, fibroblasts	Proliferation, differentiation	Specific blockers; gene knockout	Arrhythmia, hypertrophy, heart failure	[38,164,171,172]
TRPV1	Fibroblasts		Specific activators and inhibitors	Hypertrophy, heart failure	[173,174,175,176,177]
TRPV2	Myocytes, fibroblasts	Differentiation	Inhibitors; gene knockout	Dilated and ischemic cardiomyopathy	[178,179,180,181]
TRPV3	Myocytes, fibroblasts	Proliferation	Activators	Hypertrophy	[182,183]
TRPV4	Myocytes, fibroblasts	Differentiation	Inhibitors; gene knockout	Myocardial infarction	[39,184,185]
TRPM2	Myocytes, fibroblasts		Gene knockout	Ischemic injury	[186,187,188,189]
TRPM7	Myocytes, fibroblasts	Proliferation, differentiation	shRNA	Cardiac fibrosis	[37,72,190,191,192,193,194,195,196]
TRPA1	Myocytes, fibroblasts	Differentiation	Inhibitors, gene knockout	Hypertrophy, heart failure	[197,198,199,200]
